# Macro- and Trace-Element Intake from Human Milk in Australian Infants: Inadequacy with Respect to National Recommendations

**DOI:** 10.3390/nu13103548

**Published:** 2021-10-09

**Authors:** Sabrina H. Bilston-John, Ardra Narayanan, Ching T. Lai, Alethea Rea, John Joseph, Donna T. Geddes

**Affiliations:** 1 School of Molecular Sciences, The University of Western Australia, 35 Stirling Highway, M310 Crawley, Perth 6009, Australia; sbil4512@uni.sydney.edu.au (S.H.B.-J.); ardranarayanan1994@gmail.com (A.N.); ching-tat.lai@uwa.edu.au (C.T.L.); 2 School of Mathematics and Statistics, Murdoch University, Perth 6150, Australia; Alethea.Rea@murdoch.edu.au; 3 Clinical Biochemistry and Pharmacology & Toxicology, PathWest, QEII Network, Perth 6009, Australia; John.Joseph@health.wa.gov.au

**Keywords:** lactation, trace element, macro-element, human milk, breastfeeding, minerals, intake

## Abstract

Longitudinal variations of macro- and trace elements in human milk (HM) are not well characterised, and therefore, the recommendations for intake for Australian infants require more evidence to ensure accuracy. We aimed to investigate the longitudinal variation of HM macro- and trace-element concentrations (1–12 months) and infant intake (1–6 months) and to investigate the relationships between intake and infant growth parameters at 3 and 6 months, and determine if intake was sufficient when compared to national guidelines. HM samples were collected monthly for the first 6 months and then at 9 and 12 months postpartum from mother–infant dyads (*n* = 83). Test–weighing was used to determine the volume of HM consumed daily. Element concentrations (Na, Ca, K, Mg, P, I, Se, Zn, Cu, Mn, Mo, and Fe) were measured using ICP-MS, and intake was calculated using the measured concentrations and the volume of HM consumed. The average intake of HM was 776.3 ± 24.0 mL for the infants. Changes in concentration from months 1 to 12 postpartum were observed for all the measured micronutrients (all *p* < 0.05). The calculated intakes of all the macro- and trace elements showed that 0% to 82% of infants met the current adequate recommendations at varying periods of lactation. The calculated macro- and trace-element intakes were below the adequate intake recommendations, suggesting that they are not reflective of healthy infant requirements. These findings suggest the need for larger studies using sensitive analytical techniques and the revision of current recommendations for breastfed infants.

## 1. Introduction

Human milk (HM) is a highly complex biofluid that is the optimal source of nutrition during infancy [1]. Providing all the necessary energy and nutrients, the complex composition of HM protects and supports infants in the early stages of development [2,3]. Containing a dynamic combination of macronutrients, micronutrients (including minerals, trace elements, macro-elements, and vitamins), and protective agents, the composition of HM is dependent on a myriad of factors including maternal diet and health, the stage of lactation, the external environmental conditions, and the time of day [4]. 

The World Health Organization (WHO) recommends exclusive breastfeeding for infants for the first six months of life, and the continuation of breastfeeding with the introduction of complementary foods up to two years of age and beyond. This exclusive breastfeeding recommendation is supported by research that shows, when compared to formula or other foods, HM is associated with the lowest risk of infant mortality due to infection and adverse health outcomes, including respiratory illnesses, necrotising enterocolitis, childhood leukaemia, and sudden infant death syndrome [3,5]. 

Macrominerals—sodium, potassium, chloride, calcium, magnesium, and phosphorus—and trace elements—iodine, selenium, zinc, copper, manganese, molybdenum, and iron—are all required for healthy function. Although needed in only small amounts (mg and µg), deficiencies can lead to a range of adverse health outcomes [6].

Nutritional recommendations pertaining to breastfeeding in Australia include mean macromineral and trace-element intakes for infants based on the mean concentrations of the components in milk. Due to the assumption that HM from a healthy mother provides adequate nutrition for an infant, in addition to the lack of evidence regarding the relationship between macro- and trace-element intake and infant growth, the recommended intakes of macro- and trace elements for a growing infant in Australia are based on population averages [7] known as Adequate Intake (AI) values, and these recommendations set by the National Health and Medical Research Council (NHMRC) are based on milk averages from “healthy mothers” [8]. The AI values for infants aged 7–12 months are extrapolated from estimates derived for infants less than 6 months old [8].

Infant intake recommendations are currently based upon weak evidence; comprising a few studies with small sample sizes, non-Australian cohorts, and, surprisingly, no measurement of actual intake. This is further complicated by interindividual variation between women seen in the concentrations of HM components. Unfortunately, the lack of studies pertaining to long-term (12 months) changes confound the estimations of adequate intake for infants. Indeed, some macro- and trace elements decrease rapidly in the first few months of lactation, yet the recommendations for infant intake are constant for the first 6 months of life [8,9]. Thus, investigation into the temporal variance of the macro- and trace-element intake from human milk of Australian mother–infant dyads in relation to optimal infant growth is crucial for setting appropriate recommendations.

The aim of this study was to determine any changes in the concentrations (1–12 months) and intakes (1–6 months) of macro- and trace elements in HM (Na, Mg, P, K, Ca, Cr, Mn, Fe, Cu, Zn, Se, and Mo) from a cohort of healthy Australian mothers and infants and compare these to recommended guidelines. In addition, the variation of intake between infants, the relationships between macro- and trace-element intake and infant growth parameters, and intake were investigated.

## 2. Materials and Methods

### 2.1. Study Participants and Study Design

A convenience sample was taken from a cohort of mothers and infants (*n* = 83; 44 females; 39 males) who were recruited in 2018 from the community and the Western Australian branch of the Australian Breastfeeding Association. The mothers were predominantly Caucasian (74 Caucasian, 6 Asian, 2 mixed, and 1 African). The mothers answered questionnaires regarding the maternal and infant characteristics. In addition, they self-reported maternal and infant health monthly from 1 to 6 months and at 9 and 12 months postpartum as well as breastfeeding status. The inclusion criteria included term delivery (≥37 weeks gestation), the absence of major pregnancy complications, good maternal and infant health, and intention to breastfeed for at least 12 months with an intent to exclusively breastfeed for the first 6 months. The maternal supplementation of micronutrients was noted but was not expected to affect the concentrations in milk [10]. Zinc supplementation during lactation: effects on maternal status and milk zinc concentrations [10,11].

The exclusion criteria included preterm birth, the birth of twins, current or previous nipple piercings, and breast surgery. The participants were not followed up after the introduction of formula within the first 6 months, as the macro- and trace-element infant intake could not be calculated. This study was approved by the Human Research Ethics Committee of The University of Western Australia (RA/4/20/4023), and all the women gave written consent.

### 2.2. Sample Collection

Single HM samples were collected monthly at 1, 2, 3, 4, 5, 6, 9, and 12 months postpartum (pp) by hand expression into 30 mL tubes. The mothers expressed from the same breast, in the morning, for the entire 12 months and refrained from breastfeeding/expressing milk from the breast for a minimum of two hours prior to expressing the sample directly into sterile tubes. However, there was one exception for which the left side was used for 1–6 months and the right side was used for 9 and 12 months for one mother. All the samples were immediately stored in home freezers before being transported on ice to The University of Western Australia (UWA) for storage at −80 °C to ensure the elemental composition of each sample was reflective of the time of collection.

A series of 24 hour milk productions were conducted at 3 months pp. The volume of HM consumed by the infant was measured using the test–weigh method as previously described [12]. The mothers were provided with electronic scales (Medela Baby Scale, Baar, Switzerland; accurate to 2 g) to weigh the infant before and following each feed. The difference in weight is considered to be equivalent to the volume in millilitres (HM: 1.03 g/mL) [13]. The daily intake was calculated as the sum of the sum of all the feeds in 24 hours. This intake volume was used in the calculation of the macro- and trace-element intakes at 1–6 months, as normal established lactation occurs by 2–4 weeks pp and remains constant [14,15].

### 2.3. Analytical Methods

Samples were prepared and analysed according to the method outlined in Bilston-John et al. 2021 [16]. In brief, the samples were diluted using a tetramethylammonium hydroxide (TMAH) solution containing internal standards, yttrium, scandium, gallium, and indium. Calibration samples were prepared from commercially available standards (O2si Smart Solutions^®,^ North Charleston, SC, USA). Quality control samples were prepared from commercially available standards to ensure accurate analysis in every batch (Recipe Chemicals GmbH, Bio-Red Laboratories Inc., Hercules, CA, USA). The PlasmaQuant MS Elite paired with the ASpect MS data analysis system (Analytik Jena AG, Jena, Germany) was used for analysis, and details of the instrument operating conditions are shown in Supplementary Table S1. Signal drift was prevented by analysing samples in batches of 45, performing additional calibration, using quality control samples, and continually monitoring the measured concentrations of the internal standards.

### 2.4. Infant Growth Parameters

The weights, lengths, and head circumferences of the infants were collected at 3 and 6 months postpartum. The z-scores for each of the anthropometric variables— length-for-age (LFA), head circumference-for-age (HCFA), and weight-for-age (WFA)—were calculated using the WHO child growth standards [17].

### 2.5. Statistical Analysis

The data were analysed using IBM^®^ SPSS^®^ Statistics (Version 26.0. Armonk, NY, USA: IBM Corp), and Prism 8.4.3 (Version 471, GraphPad Software, San Diego, CA, USA) was utilised to produce the graphs. The data are presented as the means ± standard errors of the mean (SEMs) unless stated otherwise. The daily intakes of macro- and trace elements were calculated using the measured concentrations of each nutrient in HM and daily volume of milk consumed as determined by the test–weigh method, according to the following formula:

(1) $$Infant\;intake\;\left(\frac{mg\;or\;\mu g}{day}\right)=Concentration\;micronutrient\;\left(\frac{mg\;or\;\mu }{L}\right)\times 24hour\;milk\;intake\;\left(L\right)$$

The changes in macro- and trace-element concentrations over the first 12 months pp and changes in intake over the first six months pp were determined using linear mixed modelling, with months pp fitted as fixed effects and individual mothers fitted as random effects. Bonferroni adjustments were used to account for the number of monthly comparisons. In addition, the effect of macro- and trace-element supplementation by mothers within the cohorts on the concentration in HM was analysed using linear mixed modelling. The month of lactation and individual mothers were fitted as random effects, and supplementation was fitted as a fixed effect. To assess the variation between months and between individuals, the variation due to separate individuals was compared to the residual variance in the linear mixed model. The relationships between macro- and trace-element intake and infant anthropometric variables represented as z-scores to account for sex differences were determined using multiple regression analysis. Assumptions of linearity, normality, and the absence of collinearity were determined using patrial regression plots, Q–Q plots, and the assessment of tolerance values > 0.1, respectively. Where assumptions were met, the significance of the linearity between variables was determined by applying a Bonferroni adjustment to *p* = 0.05.

## 3. Results

### 3.1. Cohort Characteristics

Information regarding the demographics and HM collection of the cohort is shown in Table 1. Missing data included incomplete participant questionnaires and missing intakes due to problems associated with test–weighing.

### 3.2. Changes in Macro- and Trace-Element Concentrations over the First 12 Months Postpartum

Due to limited sample volumes or the absence of samples, the missing samples were 9 at 1 month, 11 at 2 months, 10 at 3 months, 17 at 4 months, 26 at 5 months, 23 at 6 months, 34 at 9 months, and 54 at 12 months. There was no difference in the concentration of calcium (*p* = 0.239), magnesium (*p* = 0.665), iron (*p* = 0.723), or zinc (*p* = 0.573) in HM in mothers taking supplements compared to those not taking supplements.

**Figure 1:**
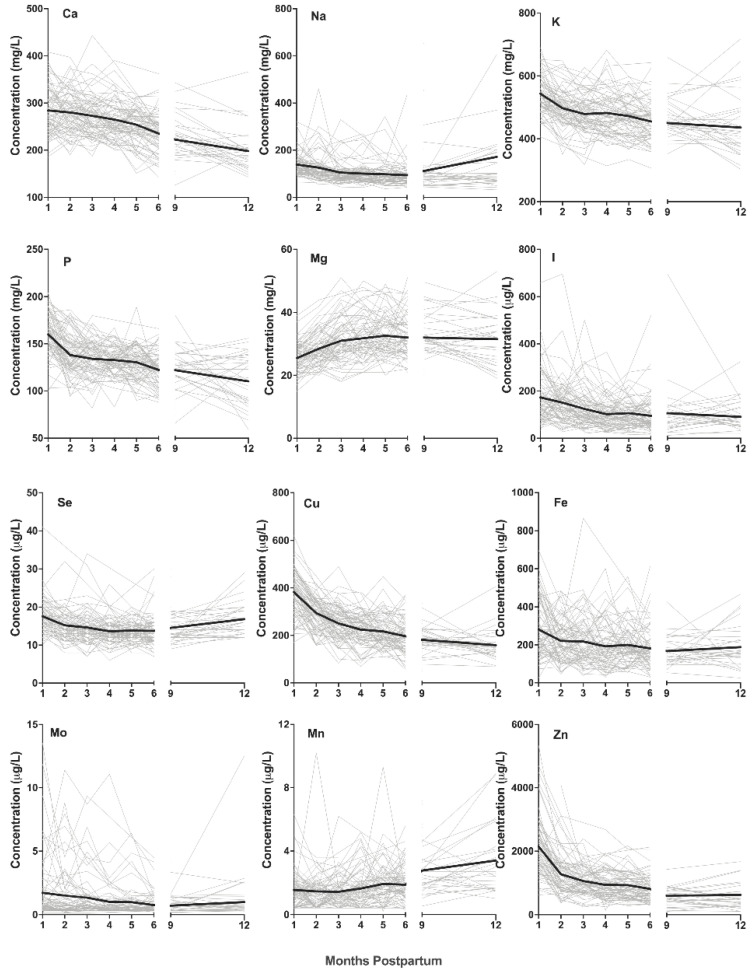
Macro- and trace elements in human milk over the first 12 months postpartum.

The calcium concentrations decreased over the first 12 months of lactation (Figure 1), with the HM calcium in the first month significantly higher than that at 5, 6, 9, and 12 months (*p* < 0.001 for all comparisons) pp. HM sodium did not change during the first 3 months of lactation (*p* > 0.05), but months 1–2 were significantly higher than months 3–6 (*p* < 0.001 for comparisons) (Figure 1). There was an increase in HM sodium at 12 months compared to 3–6 months pp (*p* < 0.001). The HM potassium concentration was the highest at 1 month pp than any other time periods (*p* < 0.001 for all comparisons) (Figure 1), and the concentration at 9 and 12 months was lower than that for 1–4 months (*p* < 0.05 for all comparisons). The concentration of phosphorus was the highest in the first month pp (*p* < 0.001 for all comparisons) (Figure 1), and the concentration decreased at 6 months (*p* < 0.05 compared to 1–4 months). The HM magnesium was the lowest in the first month pp (*p* < 0.01 for all comparisons) (Figure 1). The concentration at 2 months was lower than that at months 3–9 (*p* < 0.05 for all comparisons) but not significantly different at 12 months (*p* = 0.15). The HM iodine in the first month was higher than that at all other times (*p* < 0.001 for all comparisons), except for 2 months (*p* = 1.0) (Figure 1). The HM selenium was the highest at 1 month (*p* < 0.01 for 2–9-month comparisons) except for the comparison at 12 months, for which there was no difference (*p* = 1.0) (Figure 1). The HM copper was the highest at 1 month (*p* < 0.001 for all comparisons) (Figure 1). The concentration at 6 months was lower than that for 1–3 months (*p* < 0.001 for all comparisons). The HM manganese did not vary over the first 6 months pp (*p* = 1.0 for all comparisons); however, the concentrations at 9 and 12 months were significantly higher than those at 1–6 months (*p* < 0.001 for all comparisons) (Figure 1). The HM molybdenum was higher at 1 month pp than the concentrations at 4–9 months (*p* < 0.05 for all comparisons) (Figure 1). The HM iron was the highest at 1 month pp (*p* < 0.05 for all comparisons), and the concentrations from 2 to 12 months did not vary (*p* > 0.05 for all comparisons) (Figure 1). The HM zinc decreased steadily over 12 months pp (Figure 1). The zinc concentration was the highest at 1 month pp (*p* < 0.001 for all comparisons), and that at 12 months pp was lower than that for months 1–5 (*p* < 0.05 for all comparisons) (Figure 1).

The grey lines indicate individual plots; the black lines indicate mean values. Abbreviations: Se, selenium; Cu, copper; Mn, manganese; Mo, molybdenum; Fe, iron; Zn, zinc; Ca, calcium; Na, sodium; K, potassium; P, phosphorus; Mg, magnesium; I, iodine. The selenium and manganese concentrations increased in months 9 and 12. The copper decreased from 1 to 12 months. The molybdenum concentrations were the highest at months 1 and 2 and remained stable for the duration of lactation. The iron concentration was the highest in the first month and remained stable thereafter. The zinc decreased from 1 to 9 months. The calcium and potassium concentrations decreased from 1 to 6 months. The sodium and phosphorus concentrations decreased from 1 to 6 months and increased in months 9 and 12. The magnesium concentration increased from 1 to 3 months and remained stable thereafter. The iodine concentration was the highest in months 1 and 2 and did not change in months 3–12.

In summary, the elements magnesium, potassium, phosphorus, copper, iron, and zinc were all the highest in the first month pp, and the elements calcium, iodine, selenium, sodium, and molybdenum were higher in the first three months pp compared to months 4–6. The concentration increased in late lactation for the elements selenium, manganese, and sodium.

### 3.3. Changes in Macro- and Trace-Element Intake over the First Six Months Postpartum

The median intakes of 12 macro- and trace elements over the first 6 months pp are shown in Table 2, alongside the recommended AI values.

**Figure 2:**
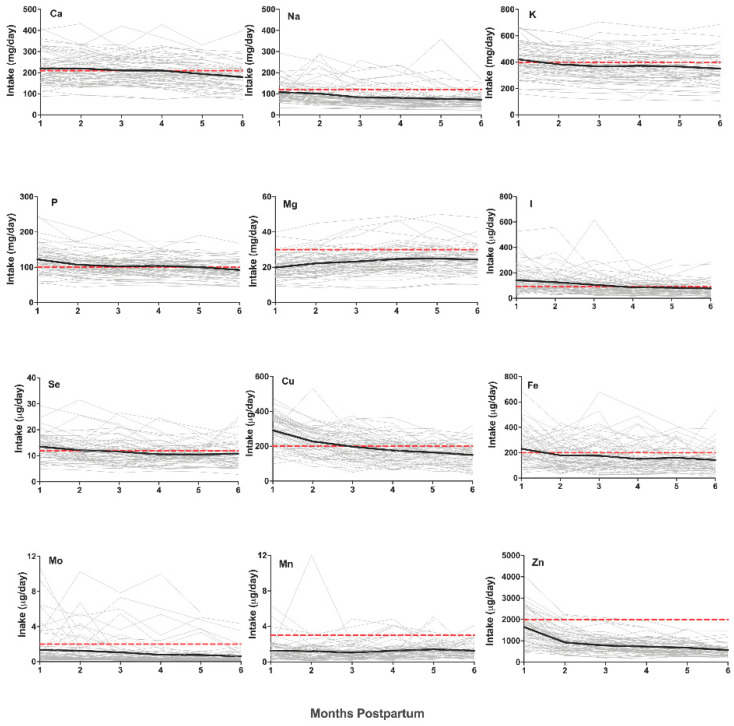
Intake of macro- and trace elements in human milk over the first 6 months postpartum.

The intake of calcium declined over the first 6 months and was lower at 6 months than months 1–4 (*p* < 0.001 for all comparisons). The mean calcium intake fell below the AI value after 4 months and remained below the AI for months 5 and 6 (Figure 2).

The intake of sodium declined over the first 6 months, and the intake at 1 month was higher than that at months 3–6 (*p* < 0.001 for all comparisons). The mean sodium intake was below the AI value for all the recorded monthly intervals (Figure 2).

The potassium intake decreased across the first 6 months pp, with the first month having the highest intake compared to all the other months (*p* < 0.001 for all comparisons). The mean intake of potassium was above the AI in the first month but fell below for all the remaining months (Figure 2). The phosphorus intake decreased throughout the first six months, with the first month having the highest intake compared to all the other months (*p* < 0.001 for all comparisons). The mean intake was above the AI value for the first 5 months pp but was below the AI in the sixth month (Figure 2). The intake of magnesium was the lowest in the first month (*p* < 0.01 for all comparisons), and the mean intake was insufficient when compared to the AI at all the time points (Figure 2).

The intake of iodine decreased over the first 6 months pp and was higher in the first month compared to months 3–6 (*p* < 0.01 for all comparisons). Compared to the recommended AI, the mean intake was sufficient in the first 3 months but fell below the AI for months 4–6 (Figure 2).

The grey lines indicate individual plots, the black lines indicate mean values, and the dashed red line shows the AI recommendation. Abbreviations: Se, selenium; Cu, copper; Mn, manganese; Mo, molybdenum; Fe, iron; Zn, zinc; Ca, calcium; Na, sodium; K, potassium; P, phosphorus; Mg, magnesium; I, iodine. Fewer than 50% of the infants met the AI recommendation for selenium by 2 months. Fewer than 50% of the infants met the AI recommendation for copper or iodine by 3 months. For manganese and molybdenum, the largest proportion of infants met the AI recommendation at 4 months, 10.5%, and 2 months, 16.9%, respectively. Fewer than 50% of the infants met the AI recommendation for iron or calcium at any point. By 3 months, no infants met the AI recommendation for zinc. Fewer than 23% of the infants met the recommended sodium intake at any time point. Fewer than 50% of the infants met the recommended potassium or phosphorus intake by 2 months and thereafter. For magnesium, the largest proportion of infants met the AI recommendation at 5 months, 25%.

The intake of selenium was the highest in the first month (*p* < 0.01 for all comparisons) and decreased throughout the first six months pp. The mean intake relative to the recommended AI was sufficient in the first month but insufficient for all the months thereafter (Figure 2).

The copper intake was the highest in the first month (*p* < 0.001 for all comparisons) and decreased throughout the period of lactation, with the intake at 6 months being lower than that at months 1–3 (*p* < 0.001 for all comparisons). The mean intake was above the recommended AI for the first 3 months pp but fell below for months 4–6 (Figure 2). The intake of manganese did not vary throughout the first six months pp (*p* > 0.05 for all comparisons). In addition, the mean intake was lower than the recommended AI at all the recorded time points (Figure 2).

The molybdenum intake decreased throughout the first 6 months, and the intake in the first month was higher than that at months 4 and 6 (*p* = 0.048 and *p* = 0.002, respectively). The mean intake was insufficient at all the months when compared to the recommended AI (Figure 2).

The intake of iron was the highest in the first month pp (*p* < 0.05 for all comparisons) and did not vary from months 2 to 6 (*p* > 0.05 for all comparisons). The mean intake was higher than the recommended AI in the first month but insufficient for all the months thereafter (Figure 2).

The zinc intake was the highest in the first month pp (*p* < 0.001 for all comparisons) and decreased over 6 months, with the intake at 6 months lower than that at 1–3 months (*p* < 0.05 for all comparisons). The mean intake compared to the recommended AI was insufficient at all the time points (Figure 2).

In summary, the intake decreased over the first 6 months pp for the elements calcium, phosphorus, potassium, iodine, copper, molybdenum, sodium, selenium, and zinc. The intake of iron was the highest at 1 month pp but did not change thereafter. The intake of manganese did not change, and the intake of magnesium increased over the course of lactation. The intakes of the elements sodium, magnesium, manganese, molybdenum, and zinc were insufficient compared to the AI recommendation at all the time points. After the first month, the mean intakes of iron, selenium, and potassium were below the AI recommendations. The iodine and copper mean intakes fell below the AI at 4 months pp and remained below thereafter. The mean intake was insufficient for calcium at 5 months and phosphorus at 6 months pp.

### 3.4. Relationship between Intake and Infant Growth Parameters

There was no association between any anthropometric outcome and the intake of any macro- or trace elements at 3 or 6 months pp (*p* > 0.05). There was also no association between the change in weight or length z-score from birth to 6 months and the average intake of macro- or trace elements over the first 6 months of life (*p* > 0.05).

## 4. Discussion

The majority of the macro- and trace elements measured showed the highest concentrations in the first month of lactation, with the exception of magnesium. The physiological mechanisms that govern high secretion are unknown but suggest a potential response to high requirements in early infancy. Importantly, the intakes in this study were substantially lower than those in previous studies [18,19], and higher than those in studies associating HM macro- and trace-element intakes with infant infection and reduced growth [20,21], suggesting that the healthy intake levels fall within a wide range. The variation between studies is likely a combination of the variation between individual mothers, different sampling protocols, assumptions versus measurements of HM volume, and analytical techniques.

The zinc concentration (2146 µg/L) and intake (1527 µg/day) are high in the first month of lactation and gradually decline over the course of lactation. Whilst a decline in concentration indicates the depletion of maternal stores and thus decreased availability for secretion into HM, previous studies report no impact of maternal intake on HM concentration, thus suggesting that secretion into HM is tightly regulated [22]. Zinc deficiency is prevalent worldwide and is considered a major risk factor for the global burden of disease [23]. Physiologically, zinc plays an important role in preventing infection and supporting optimal infant growth [24] and is prevalent in developing countries, where infant zinc deficiency is high. Osendarp et al. [20] supplemented Bangladeshi infants aged 4 to 24 weeks and showed improved growth rates and a reduced incidence of acute lower respiratory infections; however, this improvement was only observed when the initial infant plasma zinc levels were below 9.18 mol/L. This low plasma zinc is likely due to inadequate zinc stores at birth as hepatic metallothionein, common in low-birth-weight infants, of which there are high rates in developing countries [25]. In this study, no relationship between zinc intake and growth was observed, which is likely due to the fact that this was a healthy, term cohort. This suggests that, once a minimum serum zinc concentration in infants is met as a result of adequate hepatic stores, HM zinc intake does not influence growth but rather protects against processes that may limit it. Furthermore, the infant intake of zinc was well below the recommended AI for all the infants in this study by 3 months, indicating the current AIs are higher than the requirements for healthy infants or that the AI should not encompass a full 6-month period.

The iodine concentration and intake were the highest at 1 month pp and remained constant from months 3 to 12 (Figure 1), consistent with previous studies [26]; however, there are a few reports of decreasing levels in the first month, most likely due to inconsistencies of sampling for the lactation stage. Furthermore, we found that the intake of iodine fell below the AI value by 4 months of age, suggesting that single recommendations do not accommodate changes across time. As the infants in this study were healthy and reported no symptoms of iodine deficiency, our data suggest that these concentrations and subsequent intakes of iodine are more representative of the amounts of iodine needed to meet physiological demands during the early stages of life.

The selenium concentrations decreased from months 1 to 6 and then increased at 12 months pp (Figure 1). HM selenium is affected by maternal intake, and the concentrations in maternal blood are 10-fold those of HM [27]. The selenium intake was below AI recommendations at 2 months pp. As an increased maternal intake of selenium has been linked to improved infant intake in previous studies, it is reasonable to assume that greater dietary selenium would improve the proportion of infants meeting this requirement [28,29]. However, as no correlation between selenium intake and growth was found, and the infants in this cohort were healthy, it is likely that this is more representative of a healthy intake range, and caution should be taken in recommending maternal supplementation.

The stable concentration of magnesium from months 3 to 12 found in this study parallels published literature (Figure 1) [30]. However, a few studies have shown an increase in concentration from 1 to 3 months as reported in this study (18.4–22.1 µg/L) [31,32]. As magnesium is important for bone development, this rise may be due to the rapid increase in bone density that occurs in the first 6 months [33]. The mean magnesium intake was less than the recommended AI at all the stages of lactation. It has been previously reported that intestinal magnesium absorption is dynamic and increases in response to low intake [30]. Whilst this has not been specifically demonstrated in infants, this mechanism and the lack of correlation between growth and magnesium intake suggest that the intake in this cohort was sufficient to meet physiological demands.

The calcium and phosphorus concentrations were the highest in the first month and decreased over the 12 months of lactation (Figure 1). In addition to magnesium, calcium and phosphate are crucial in bone mineralisation; however, the concentrations of both elements decreased in months 1–3 pp, contrasting with the described climb in magnesium [34]. Whilst no measurements on bone density were collected, the normal growth trajectories of the infants in this study suggest that the intakes of calcium and phosphorus were adequate for growth despite the mean intake being below the AI recommendation for calcium by 4 months and phosphorus in the sixth month.

The molybdenum concentration was the highest in the first 2 months pp and remained stable for the duration of lactation (Figure 1). Knowledge of the molybdenum status in infants and mothers during lactation is extremely limited. The influence of maternal intake on the molybdenum HM concentration has not been characterised; however, the variability between individual mothers suggests that external factors such as diet may affect concentrations. The concentration and intake of molybdenum recorded in this study were very low, and no associated symptoms in infants were observed that could be explained by inadequate molybdenum intake. However, previous supports suggest that infants’ molybdenum stores are seven times less than adults’ and, thus, their requirements must be met by intake [35]. The defining of molybdenum as an essential trace element is due to studies in other animals showing impaired ossification associated with low molybdenum; however, no deficiency in human infants has yet been reported [36].

The copper concentration and intake decreased over the course of lactation, consistent with previous studies (Figure 1; Table 2) [37,38]. The decline in intake resulted in the mean intake falling below the recommended AI by 4 months pp. Infants are born with hepatic copper stores and, similar to the case for zinc and iron, the healthy, term infants studied here combined with the lack of correlation between intake and growth suggest that HM copper was sufficient to support the physiological needs for growth; thus, revised infant AIs should be considered [39].

The iron concentration was the highest in the first month pp, after which it remained constant for the duration of lactation (Figure 1). The iron concentrations found here were lower than those in previous studies [18,19]; however, the lack of correlation between iron intake and growth suggests that this was not growth limiting. Whilst the low concentration may suggest secretion is an unregulated process, the expression of ferroportin, a transmembrane protein believed to be a primary transporter of iron into HM, parallels the change in Fe concentration over the course of lactation, suggesting that secretion into HM is controlled [39]. Infant hepatic iron stores are reported to be high, raising pertinent questions about the need for supplementary iron in breastfed infants [39]. Studies of formula-fed infants have identified iron deficiency in a proportion of infants, which is likely due to the poor bioavailability of iron, the association of iron with the milk fat globule, and the possible compounding factor of delayed cord clamping not being routine during the birth [39,40].

In this healthy cohort, no relationships between infant growth parameters and the intakes of any macro- or trace elements were identified, suggesting that the relationship between macro- and trace-element intake and growth is non-linear and that thresholds may exist below which clinical deficiency manifests. Therefore, recommendations need to be considered in the context of intakes associated with proven deficiency. Consistent with this threshold theory, Umeta et al. [21] demonstrated that zinc supplementation improved growth in stunted infants, and Rivera et al. [41] reported similar findings with the supplementation of iodine, selenium, iron, zinc, copper, and manganese. The healthy nature of our cohort and normal growth of the infants suggest that a combination of infant health, infant stores during gestation, and intake from HM led to adequate macro- and trace-element statuses in infants. However, in populations where maternal health is poor and infants are born with low macro- and trace-element stores, supplementation may be required. Therefore, a reduction in the Australian AI recommendations with consideration of the environmental factors of this population when determining if intake from HM is adequate should be considered in the future.

The wide variation in the concentrations and intake of all the macro- and trace elements between mothers and infants also suggests the need for recommended AI ranges. The variation in intake was large, suggesting that individual volumes did not reduce the variability that was seen for concentration. This variation suggests that individual intake must be accounted for in the calculation of daily macro- and trace-element intake, as opposed to the application of a constant 780 mL intake or a mathematical intake estimation with no validation. The establishment of recommended ranges, such as those used in assessing healthy amounts of nutrients in blood, is needed to account for the intake variation of healthy infants, so as to prevent the incorrect diagnosis of deficiency and unnecessary supplementation.

Whilst high AI recommendations may not immediately appear to be problematic, they can potentially lead to unnecessary supplementation and have a negative impact on breastfeeding. For example, iron supplementation in infants from Sweden and Honduras was associated with higher rates of diarrhea than those in a placebo group, suggesting that supplementation may have toxicity-associated complications [40]. Furthermore, intakes of macro- and trace elements during the period of exclusive breastfeeding that are deemed to be too low may lead to the early introduction of complementary foods. Not only would this reduce HM intake during a time when exclusive breastfeeding has been shown to be the best nutritional source, but complementary foods can lead to the decreased bioavailability of HM macro- and trace elements such as iron, further limiting their uptake and absorption [42]. This highlights that the assumption that infant intake below the AI recommendation should be improved does not necessarily lead to positive health outcomes.

The decline in HM macro- and trace-element intake and concentration seen in this study supports the suggestion for more time-specific AI recommendations. Furthermore, the variation in intake reported between studies with healthy infants negates the idea that a single intake value is representative of what is considered adequate. Therefore, we propose the need for the introduction of AI ranges as opposed to minimums, and the separation of the first 6 months pp into 1–3 and 4–6 intervals to more accurately reflect the changing nature of macro- and trace-element intake. As discrete lower bounds at which intakes transition from low intakes to inadequate are not fully elucidated, further research will be needed to establish the point at which reduced intake has clinical consequences.

### Limitations and Further Directions

Although infant weight, length, and head circumference were considered with relation to macro- and trace-element intake, the growth of infants can be defined by a myriad of variables such as fat mass and fat-free mass, which were not considered in this study. Furthermore, the additional consideration of non-physical developmental outcomes may provide insight into relationships between cognitive development and macro- and trace-element intake. Previously, studies have determined the absorption of macro- and trace elements from HM into infants’ bloodstreams; however, not all the elements discussed in this study have been thoroughly investigated. Thus, more conclusive results between macro- and trace elements in HM and their relation to the developmental outcomes of infants could be established through the testing of infant serum in parallel with HM intake to take into account the degree of absorption of macro- and trace elements rendering them available for physiological processes.

The processes by which macro- and trace elements are secreted into human milk are not well understood. Whilst an understanding of the specific transport mechanisms for individual elements has been postulated in recent years for copper, zinc, and iron [39], for other elements including those for which the health benefits are still not well-understood, such as manganese, there is an absence of literature regarding any specific transporter proteins or pathways. Future research surrounding the molecular mechanisms governing secretion into human milk will provide a robust understanding of the micronutrient composition of human milk.

The wide variability of the reported macro- and trace-element intakes from HM supports the need to establish a long-term study with a large cohort of Australian mothers and infants with the aim of monitoring developmental outcomes and their relation to macro- and trace elements in HM. Whilst efforts are being made globally to design studies with the purpose of generating more informed RDI values such as that proposed by Allen and Hampel [43], individual measurements of the HM volume consumed are rarely accounted for. The findings presented here suggest that these future studies will still be limited if individual intake is not considered. A study encompassing the collection of HM and maternal and infant blood in addition to 24-hour milk intake profiles is necessary to provide more substantiated recommendations, with the aim of better informing the health of the growing Australian infant.

## 5. Conclusions

This study of Australian breastfed infants shows that macro- and trace-element intake from human milk generally decreases in the first 6 months postpartum. Furthermore, the measured infant intakes in this study were commonly lower than the current recommended intakes for all the macro- and trace elements measured in the healthy breastfed infants. Larger studies are required to confirm these results, and the revision of adequate intake recommendations from national guidelines is required.

## Figures and Tables

**Table 1:** Demographic characteristics of the cohort. Data shown as mean ± SEM with given ranges where appropriate, unless stated otherwise.

Characteristic	Mean ± SEM	Range
Maternal age at infant birth (*n* = 81)	33.2 ± 0.52	25.1–46.4
Parity (*n* = 83)	2.1 ± 0.1	1–4
Maternal ethnicity (*n* = 83)		
Caucasian	74	
Other	9	
24-hour infant breast milk intake (mL, *n* = 59)	776.3 ± 24.0	348–1344
Maternal supplementation (*n* = 50) Calcium Magnesium Iron Zinc	*n* = 10 *n* = 18 *n* = 38 *n* = 8	
HM collection^+^ (*n* = 83)		
Left breast/right breast	29/54	
Infant sex (*n* = 83)		
Male/female	39/44	
Gestational age (weeks)	39.2 ± 0.1	36.6–41.2
Infant birth weight (kg) (*n* = 78)	3.49 ± 0.48	2.36–4.36
Infant WFA z-score at birth (*n* = 78)	0.38 ± 0.1	−2.08–2.16
Infant length at birth (cm) (*n* = 78)	51.0 ± 0.28	45–59
Infant LFA z-score at birth (*n* = 78)	0.78 ± 0.15	−2.23–4.82

^+^ Samples were collected from the same breast side at every time interval, except for one mother, for which the left side was used for 1–6 months and the right side was used for 9 and 12.

**Table 2:** Median intakes of macro- and trace elements at 1–6 months pp compared to recommended AI (Adequate Intake) values. Data shown as median (range) for measured intakes and number (percentage) for infants meeting the AI. Infant intake expressed as median (range). Infants meeting AI expressed as *n* (%).

		1 Month (*n* = 74)		2 Months (*n* = 72)		3 Months (*n* = 73)		4 Months (*n* = 66)		5 Months (*n* = 57)		6 Months (*n* = 60)	
	AI Value	Intake Median (Range)	Meeting AI *n* (%)	Intake Median (Range)	Meeting AI *n* (%)	Intake Median (Range)	Meeting AI *n* (%)	Intake Median (Range)	Meeting AI *n* (%)	Intake Median (Range)	Meeting AI *n* (%)	Intake Median (Range)	Meeting AI *n* (%)
Ca (mg/day)	210	201 (87.6–411)	28 (45.2)	204 (93.6–431)	27 (45.8)	195 (83.6–421)	27 (44.3)	195 (74.1–427)	24 (42.1)	190 (86.1–330)	18 (34.6)	175 (74.5–399)	15 (29)
Na (mg/day)	120	95.7 (34.5–293)	14 (22.6)	85.5 (31.3–288)	13 (22.0)	73.5 (38.2–259)	9 (15.0)	67.0 (26.2–238)	9 (15.5)	64.6 (24.5–358)	6 (11.5)	67.7 (23.4–168)	4 (7.80)
K (mg/day)	400	418.1 (160–673)	33 (53.2)	376 (132–624)	26 (44.1)	365 (119–704)	21 (34.4)	372 (109–673)	22 (38.6)	362 (116–638)	16 (30.8)	326 (116–638)	15 (29.4)
P (mg/day)	100	121 (50.4–246)	44 (71.0)	100 (42.8–211.9)	29 (49.2)	97.9 (48.4–206)	27 (44.2)	104 (47.3–173)	32 (56.1)	98.0 (44.9–191)	24 (46.2)	87.5 (44.6–168)	17 (32.7)
Mg (mg/day)	30	18.6 (9.13–39.8)	3 (4.84)	22.1 (8.35–44.9)	9 (15.3)	23.8 (8.03–49.0)	7 (11.5)	24.3 (8.03–49.0)	12 (21.1)	24.3 (10.2–50.1)	13 (25)	22.9 (10.1–48.0)	9 (17.3)
I (µg/day)	90	119 (26.0–528)	37 (59.7)	109 (25.1–557)	35 (59.3)	88.6 (21.8–617)	29 (47.5)	65.6 (14.5–303)	21 (37.5)	72.1 (21–304)	16 (31.3)	54.6 (8.82–286)	15 (29.4)
Se (µg/day)	12	13.3 (4.87–29.3)	36 (58.1)	11.1 (4.52–31.5)	25 (42.3)	10.8 (3.48–26.7)	24 (39.3)	10.2 (4.18–24.7)	16 (28.1)	10.1 (3.83–18.8)	14 (26.9)	10.6 (3.83–18.8)	14 (26.9)
Cu (µg/day)	300	299 (98.5–474)	51 (82.2)	218.7 (82.5–532)	37 (62.7)	195 (50.8–374)	28 (45.9)	179 (63.9–364)	20 (35.1)	157 (56.6–302)	15 (28.8)	159 (36.2–321)	10 (19.2)
Mn (µg/day)	3	1.00 (0.19–6.38)	4 (6.45)	1.00 (0.14–12.4)	3 (5.01)	0.89 (0.16–4.86)	1 (1.64)	0.95 (0.17–4.86)	6 (10.5)	1.22 (0.23–5.07)	3 (5.77)	1.03 (0.26–4.12)	3 (3.92)
Mo (µg/day)	2	0.56 (0.06–10.6)	9 (14.5)	0.42 (0.15–10.3)	10 (16.9)	0.42 (0.06–7.83)	8 (13.1)	0.31 (0.11–10.0)	4 (7.02)	0.35 (0.10–5.58)	4 (7.69)	0.30 (0.10–4.33)	2 (3.85)
Fe (µg/day)	200	185 (24.1–686)	28 (45.1)	145 (29.2–436)	22 (37.3)	138 (22.0–677)	19 (31.1)	123 (24.4–490)	14 (26.9)	138 (39.3–396)	14 (26.9)	126 (12.1–539)	11 (21.2)
Zn (µg/day)	2000	1569 (452-4058)	16 (25.8)	848 (300–2233)	3 (5.08)	768 (192–1908)	0 (0)	594 (240–1571)	0 (0)	573 (240–1471)	0 (0)	539 (203–1471)	0 (0)

## Data Availability

The data presented in this study are available on request from the corresponding author.

## Supplementary Materials

The following are available online at https://www.mdpi.com/article/10.3390/nu13103548/s1. Table S1: ICP-MS Operating conditions—PlasmaQuant MS Elite.
