# Supplementary material for: Macro- and Trace-Element Intake from Human Milk in Australian Infants: Inadequacy with Respect to National Recommendations
Source: Nutrients. 2021 Oct 9;13(10):3548. doi: 10.3390/nu13103548 (PMC8537127; doi:10.3390/nu13103548)
Supplement: Supplementary file 1 [file nutrients-13-03548-s001.zip › nutrients-1382923-supplementary.pdf]

Supplementary Table 1. **ICP-MS Operating conditions – PlasmaQuant MS Elite**

Flow Parameters (L/min)	
Plasma Flow	9.0
Auxiliary Flow	1.5
Sheath Gas	0.10
Nebulizer Flow	0.93
Sampling depth (mm)	7.0
RF Power (kW)	1.30
Pump rate (rpm)	14
Sample uptake delay (s)	8
Stabilisation delay (s)	35
Rinse time (s)	10
Scans/Replicate	10
Replicates/Sample	5
Scan Mode	Peak hopping
Acquisition Mode	Steady-State
Spray chamber temperature (°C)	3
Reaction gas	H_2 for all elements
Reaction gas flow (mL/min)	120
Measured isotopes	Na 23, Mg 24, Al 27, P 31, K 39, Ca 44, Zn 66, Cr 52, I 127, Se 78, Cu 63, Cu 65, Mn 55, Mo 98, Fe 56
Measured internal standards	Sc 45, Y 89, In115, Ga69
